# ESOMIR: a curated database of biomarker genes and miRNAs associated with esophageal cancer

**DOI:** 10.1093/database/baad063

**Published:** 2023-10-10

**Authors:** Asma Sindhoo, Saima Sipy, Abbas Khan, Gurudeeban Selvaraj, Abdulrahman Alshammari, Mark Earl Casida, Dong-Qing Wei

**Affiliations:** Department of Bioinformatics and Biological Statistics, School of Life Sciences and Biotechnology, Shanghai Jiao Tong University, Dongchuan Road Minhang District, Shanghai 200240, PR China; Sindh Madressatul Islam University, Karachi, Sindh 74600, Pakistan; Department of Bioinformatics and Biological Statistics, School of Life Sciences and Biotechnology, Shanghai Jiao Tong University, Dongchuan Road Minhang District, Shanghai 200240, PR China; State Key Laboratory of Microbial Metabolism, Shanghai–Islamabad–Belgrade Joint Innovation Center on Antibacterial Resistances, Joint Laboratory of International Cooperation in Metabolic and Developmental Sciences, Ministry of Education and School of Life Sciences and Biotechnology, Shanghai, Minhang 200030, PR China; Centre for Research in Molecular Modelling (CERMM), Department of Chemistry and Biochemistry, Concordia University, Montreal, Quebec H4B 1R6, Canada; Department of Pharmacology and Toxicology, College of Pharmacy, King Saud University, Riyadh 11451, Saudi Arabia; Laboratoire de Spectrom´etrie, Interactions et Chimie th´eorique (SITh), D´epartement de Chimie Mol´eculaire (DCM, UMR CNRS/UGA 5250), Institut de Chimie Mol´eculaire de Grenoble (ICMG, FR2607), Universit´e Grenoble Alpes (UGA), 301 rue de la Chimie BP 53, Grenoble Cedex F-38041, France; Department of Bioinformatics and Biological Statistics, School of Life Sciences and Biotechnology, Shanghai Jiao Tong University, Dongchuan Road Minhang District, Shanghai 200240, PR China; State Key Laboratory of Microbial Metabolism, Shanghai–Islamabad–Belgrade Joint Innovation Center on Antibacterial Resistances, Joint Laboratory of International Cooperation in Metabolic and Developmental Sciences, Ministry of Education and School of Life Sciences and Biotechnology, Shanghai, Minhang 200030, PR China; Peng Cheng Laboratory, Phase I Building 8, Xili Street, Montreal, Vanke Cloud City, Nashan District, Shenzhen, Guangdong 518055, PR China

## Abstract

‘Esophageal cancer’ (EC) is a highly aggressive and deadly complex disease. It comprises two types, esophageal adenocarcinoma (EAC) and esophageal squamous cell carcinoma (ESCC), with Barrett’s esophagus (BE) being the only known precursor. Recent research has revealed that microRNAs (miRNAs) play a crucial role in the development, prognosis and treatment of EC and are involved in various human diseases. Biological databases have become essential for cancer research as they provide information on genes, proteins, pathways and their interactions. These databases collect, store and manage large amounts of molecular data, which can be used to identify patterns, predict outcomes and generate hypotheses. However, no comprehensive database exists for EC and miRNA relationships. To address this gap, we developed a dynamic database named ‘ESOMIR (miRNA in esophageal cancer) (https://esomir.dqweilab-sjtu.com)’, which includes information about targeted genes and miRNAs associated with EC. The database uses analysis and prediction methods, including experimentally endorsed miRNA(s) information. ESOMIR is a user-friendly interface that allows easy access to EC-associated data by searching for miRNAs, target genes, sequences, chromosomal positions and associated signaling pathways. The search modules are designed to provide specific data access to users based on their requirements. Additionally, the database provides information about network interactions, signaling pathways and region information of chromosomes associated with the 3ʹuntranslated region (3ʹUTR) or 5ʹUTR and exon sites. Users can also access energy levels of specific miRNAs with targeted genes. A fuzzy term search is included in each module to enhance the ease of use for researchers. ESOMIR can be a valuable tool for researchers and clinicians to gain insight into EC, including identifying biomarkers and treatments for this aggressive tumor.

**Database URL** https://esomir.dqweilab-sjtu.com

## Introduction

Esophageal cancer (EC) is one of the deadliest cancers. In 2020, EC was responsible for 0.54 million deaths with 0.6 million new cases worldwide according to Global Cancer Statistics 2020: GLOBOCAN estimation (1). Histologically, in EC, there are two subtypes: esophageal squamous cell carcinoma (ESCC) and esophageal adenocarcinoma (EAC) (2). However, it is believed that Barrett’s esophagus (BE) is the only precursor to EAC (3). Both subtypes seem very different due to distinctive primary risks, time trends, geographical patterns and genetic associations (4). The multistep process of EC development (ESCC and EAC) involves genetic occurrences that lead to important abnormalities in cell cycle control, neurotrophic interaction and adhesion molecules mechanisms (5). The occurrence of ESCC is predominant in Asian and sub-Sahara African regions, while EAC is predominant in Western countries (1).

MicroRNAs (miRNAs) are short [18–24 nucleotides (nt)] intrinsic non-coding RNAs that could control the gene transcription, i.e. pre-regulation of genes. miRNAs are a set of smallest single-stranded non-coding molecules among its other types, with 21–25 nt that regulate genes post-transcription in various species (6, 7). miRNA plays a key role in regulating biological, physiological and cellular developmental processes, including apoptosis, cell proliferation, differentiation, invasion, metastasis, metabolism, tumorigenic progression, resisting cell death and stem cell maintenance (8, 9). miRNA is the crucial player that can act as a tumor suppressor or oncogenes after being downregulated or upregulated (10). In a recent study, most miRNA gene mutations are typically found in genomic regions linked to cancer (11). Unfortunately, EC (subtypes ESCC and EAC) is diagnosed at an advanced or metastatic stage in most cases due to its asymptomatic nature, resulting in a poor prognosis and a high-death rate (12).

Recent advancement in tumorigenesis shows that molecular mechanism studies play a vital role in understanding insight into cancer (13). Molecular biomarkers are potential solutions for improving cancer invasion, metastasis and prognosis (14–16). miRNAs seem revolutionary discoveries for cancer treatment as miRNA acts as a regulator of gene expression. miRNA target identification has been a prominent area of research in recent years to gain insights into the signaling pathways, genes and mechanisms involved in cancer (17–21). miRNA prediction in vertebrates is complex as they have irregular homology in the target sequence on the seed region (22). There is substantial evidence that miRNAs play a critical role in the development of EC (23–26). miRNAs have also been utilized to classify EC based on the stage or type (27).

MiRNAs identified through computational methods and subsequently validated through experimental methods have the highest potential to alleviate the underlying mechanisms involved in cancer hallmarks. Several miRNA discoveries associated with EC networks have shown promising results for prognosis, diagnosis and treatment. There is a growing demand for a user-friendly knowledge repository containing all relevant information, including miRNAs, genes, pathways, targets, networks and more (6, 7).

Several databases are available to provide chromosomal-based information associated with targeted messenger RNA (mRNA), cancer pathways and lists of miRNAs. Some comprehensive databases, such as miRBase (28), miRGen v.3 (29) and MiRGator v 3.0 (30), provide annotated sequences, nomenclature and target information. Manually curated databases, including miRWalk (31), miRTarbase (32), miRNAMap (33) and mirRecord (34), offer experimentally validated perspectives on associated miRNAs, their interactions and targets. Additionally, there are computationally targeted prediction-based databases, such as TargetScan (35), miRDB (36), PicTar (37) and MirRabel (38). Several disease-specific databases exist to fulfill the needs of miRNA repositories, such as OncomiRdbB (39), miRCancer (40), miRactDB (41), PhenoMir (42), HDMM (43) and S-MED (44). However, these databases focus on different cancer types and provide detailed information about those diseases. Unfortunately, no detailed repository is available to provide insightful information about miRNAs involved in EC and its subtypes. Therefore, we developed and designed a comprehensive miRNA-based repository for researchers to access mRNA–miRNA targets, signaling pathways and targeted gene ontologies for EC. We created a web-based repository/database with a graphical user interface called ESOMIR (https://esomir.dqweilab-sjtu.com). Our database contains 877 human miRNAs and 133 mRNAs that act as miRNA targets in various signaling pathways, and we used MirWalk and TargetScan to cross-validate the putative targets for miRNAs. ESOMIR is a promising tool for the diagnostic paradigm in EC as it is a unique, comprehensive data repository freely accessible to everyone.

## Database design and structure

### Quarrying and categorizing miRNAs and their associated targets

**Figure 1.**
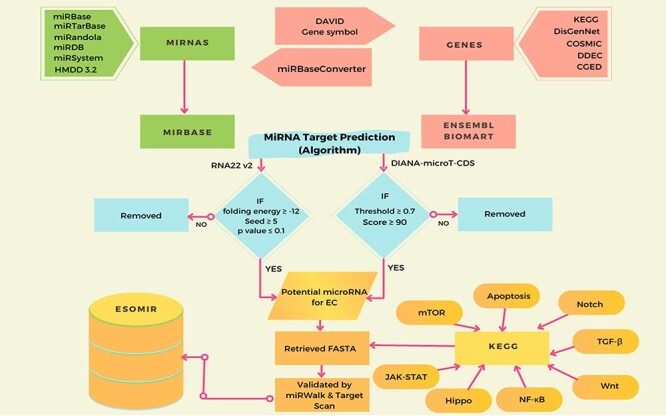
This diagram illustrates the ESOMIR modules, representing data analysis, prediction and mining procedures. Each stage in the process is clearly defined in this schematic, outlining the steps taken to reach the desired outcome.

We mined all miRNAs from literature and various databases, specifically MirTarbase, MiRDB, miRBase, MicroCosm, miR2Disease (45) and miRSystem (46). We wrote a function in Python to exclude duplicated entries and used a script to acquire all mature miRNA FASTA sequences from miRBase. We extracted the targeted sites (3′UTRs, 5′UTRs and exons) of oncogenes from literature and knowledge systems such as COSMIC (Catalogue of Somatic Mutations in Cancer) (47), KEGG (Kyoto Encyclopedia of Genes and Genomes) (48), DDEC (Dragon Database of Genes Implicated in Esophageal Cancer) (49), CGED (Cancer Gene Expression Database) (50) and DisGenNet (51). We used the Ensembl-BioMart tool (52) to download the FASTA sequence of all oncogenes. Then, we screened 2654 miRNAs against EC oncogenes using miRanda (53), DIANA-microT-CDS (54) and RNA22 v2. We identified potential miRNAs and their target sites based on the standard scores set for an appropriate target. After the screening, we used the KEGG database to find signaling pathways against each putative target. We illustrated the complete workflow from data mining to processing, prediction and validation used in the study in Figure 1.

### The structure of database and its contents

Database development is complex when providing a cognate database to users via a web-based interface with processing accuracy and speed. We developed and designed a database structure that allows users to access and extract information about miRNAs and their targets for human EC. We designed and developed ESOMIR as a cloud-based web portal to give access to in-depth analyzed data. The user interface of ESOMIR is user-friendly, which allows easy access to efficient information. We designed the structure of ESOMIR using PHP, MySQL, HTML, DataTables, Ajax, Bootstrap and jQuery.

#### Back-end development

We used the XAMPP server to design a cloud-based online portal, integrating parts such as Apache server, database (MySQL) and web interface. We developed the online portal using two scripting languages (PHP and HTML). We used MySQL and DataTables for data storage and associated operations. Additionally, we implemented logical scripts to search miRNAs, target genes or associated pathways to EC to develop the data extraction module (Figure 1). ESOMIR is directly linked to an original database, allowing users to access direct data. ESOMIR offers multiple particulars to access data associated with EC, including miRNAs, accession number, oncogene, target sites, description of genes and chromosome location.

#### Front-end development

The primary purpose of the ESOMIR interface is to provide intelligent interaction with a portal that gives the user a sense of acceptance. Ease and clarity are the foremost priorities to provide no-effort access to every group of users and enough technical assistance to do bioinformatics analysis with efficacy and effortlessness. We used Cascading Style Sheets (CSS), JavaScript, DataTables and Bootstrap to provide a modern look to this online portal.

### Selection and validation of miRNAs as putative EC miRNAs

In order to categorize potential miRNAs associated with EC, we performed prediction and analysis against all collected oncogenes using miRanda, RNA22v2 and DIANA-microT-CDS. To mine putative miRNAs, we established specific parameters for each algorithm. In miRanda, we set the analysis parameter to −15 kcal/mol energy level (EL) with a threshold of 120. The miRanda algorithm has standard settings for reasonably predicting potential target sites. The free energy of RNA molecules, which is involved in unfolding the interaction sites to allow the pairing of nucleotides between miRNAs and mRNAs, is an essential property that facilitates their interactions. Therefore, lower overall free energy means higher miRNA–mRNA complex stability, indicating a higher possibility of essential interactions.

**Figure 2.**
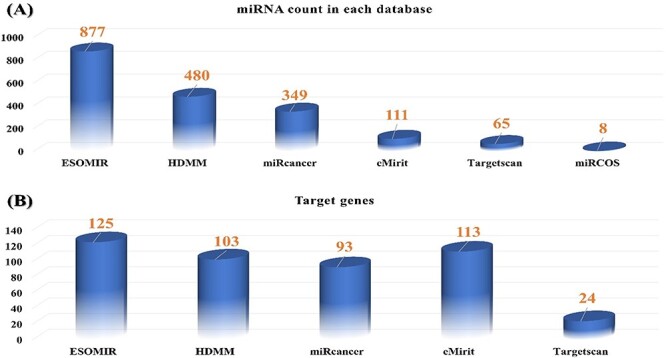
(**A**) Comparison of all available miRNA targets associated with EC in ESOMIR and other available databases. The figure shows that ESOMIR has the highest number of miRNAs included in contrast to the other available databases. (**B**) Comparison of all available mRNAs associated with EC in ESOMIR and other available databases.

For RNA22v2, we selected entities with a folding energy of −12 with a *P*-value of 0.1 and more than three seeds. Additionally, we chose a threshold of 0.7 with a score of 90 using DIANA-microT-CDS, as this is the suggested threshold range for sensitive analysis for significant results. After all selection and analysis, we identified 2028 potential miRNAs and their associated target genes and pathways for EC. We excluded data entries that did not meet the inclusion criteria and all microRNA entities that did not meet the mentioned criteria. To assess the significance of ECOMIR among other existing database systems, we plot a comparison graph in Figure 2A. We further validated the resulting miRNAs through miRWALK and TargetScan, where only experimentally validated connections were considered putative miRNAs.

### Target-based network enrichment analysis via KEGG pathways

EC is known to be regulated by different signaling pathways via microRNA, which have been shown to be significant players in the development of this disease (55–65). Using miRanda via KEGG, we identified some critical pathways in ESOMIR, including Wnt, NF-κB, TGFB, Apoptosis, Hippo, JAK-STAT and NOTCH, based on different ELs and a 120-threshold score. To ensure accuracy, we employed MirWalk and TargetScan for additional validation. In total, we identified 107 genes as miRNA targets across all signaling pathways in humans with EC. ESOMIR includes a higher number of genes as targets for EC-specific miRNAs compared to other cancer databases such as MirCosm, emiRiT (66), Mir2Disease, MirSystem, HMDD v2.0 (43) and miRCancer database (Figure 2B).

**Figure 3.**
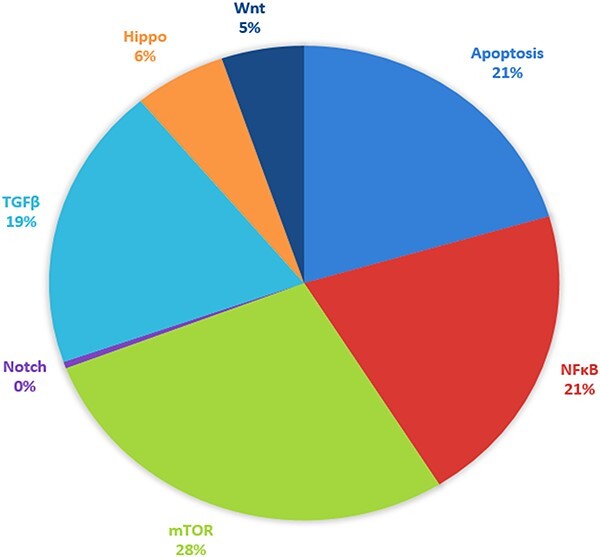
Signaling pathway targeted by selected putative miRNA associated with EC. The respective pathways along with their percentages are given and represented by different colors.

Among all signaling pathways, mTOR, apoptosis and NF-κB recorded the highest number of targeted mRNA–miRNA associated with EC, while NOTCH signaling had the least number of targets (Figure 3). A single miRNA can target one or several genes, or several miRNAs can access a single target gene. Therefore, studying all signaling pathways associated with EC, either miRNA or mRNA, can lead to exciting discoveries. Further exploration of the interaction between miRNA–mRNA via signaling pathways could provide an in-depth understanding of possible miRNA–mRNA regulation. Considering the significant role of miRNAs in regulating mRNAs in disease development, we utilized data on miRNAs and their targets in each signaling pathway to recognize their interaction. To showcase the miRNA and target interaction, we proposed a binary matrix for each signaling pathway, with 1 indicating an mRNA targeted by miRNA and 0 indicating an mRNA not targeted. We then converted the miRNA–mRNA targets data frame into a matrix to extract error-free data using an open-source Bioconductor package of R.

## Results

ESOMIR is a comprehensive database that extensively explores multiple paramount features related to EC information. The database contains a rich compilation of 125 unique genes and 877 miRNA targets implicated in EC. Accessible through a web-based portal, the database offers users access to various columns, each providing further details on the specific entity or entry under investigation. These columns include miRNA, accession, target site, EL, gene and associated information, chromosomal location and signaling pathway. Consequently, ESOMIR offers a holistic understanding of EC-related data.

### Structure of the ESOMIR database modules

**Figure 4.**
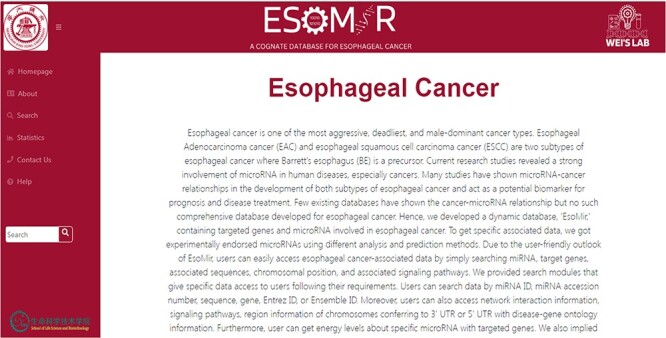
ESOMIR Homepage: a comprehensive miRNA database for EC. The home page shows the description of the database with different tabs that are included in the database. The Fuzzy Search Functionality implemented in ESOMIR is also given.

We intentionally designed the homepage of ESOMIR to exhibit simplicity and comprehensiveness regarding EC information. Additionally, we presented abstract information about the project. The webpage comprises a header, footer, responsive side navigation bar and body. A Toggle button is incorporated to facilitate the users in showing or hiding the navigation bar. The side navigation bar serves as the primary menu, enabling users to access different options promptly. We provide five pages, including the homepage, search page, statistics page, help page and contact us page. To provide a visual representation of the home page, we illustrate Figure 4.

### Fuzzy search

On every page of ESOMIR, we provide a quick fuzzy term–based search to provide quick access to users. Users can give a minimum of three characters and get the results. Users can search in any context and retrieve any data record associated with EC. We implemented fuzzy search to ease the users who might need to search for the whole term or misspell it. This approach presents relevant results to the user. Figure 4 demonstrates the representation of fuzzy search.

### Database gateways

**Figure 5.**
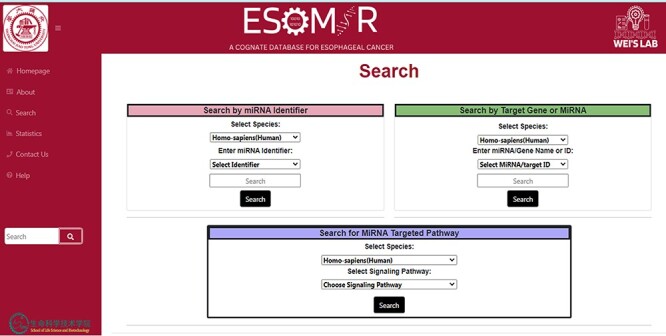
Demonstrative depiction of the Search page to access data using different modules. In the above figure from the database, it can be seen that the information can be retrieved through search by miRNA identifier search, search by target gene or miRNA or search for miRNA targeted pathway.

ESOMIR’s search page provides users with three methods to access and retrieve miRNA and their target information. The first approach involves an miRNA-based search, which offers users a selection of miRNA-related options, such as miRBase ID, accession or mature sequence. We make these options available through a drop-down menu and a search bar that users can utilize to filter or narrow their search based on specific data. The second option is gene-centric, enabling users to refine their search by selecting the gene symbol, Ensembl ID or Entrez ID. Enter the corresponding gene in the search box for specific gene data. The third and final method ESOMIR’s search page provides is interaction-network-based, which provides users with signaling pathways. The interaction-network-based method enables users to explore the signaling pathways associated with genes, miRNA and interaction by selecting pathways from a drop-down menu. Notable pathways include Wnt, NF-κB, TGFB, Apoptosis, Hippo, JAK-STAT and NOTCH. Figure 5 offers a comprehensive representation of ESOMIR’s search page.

### Upshot of data approachability

The tabular format presents the search results, including columns for miRNA, mRNA and associated information. The first column displays the miRNA and its accession number, which serves as a redirecting link to www.mirbase.org. The table also shows the target sites, indicating the predicted target site of an miRNA gene, which can be 3ʹUTR, 5ʹUTR or exon. Additionally, there is a column that provides information on ELs in kcal/mol. The respective columns illustrate the associated pathways. Targeted gene information is available through a link that redirects to GenAtlas.

**Figure 6.**
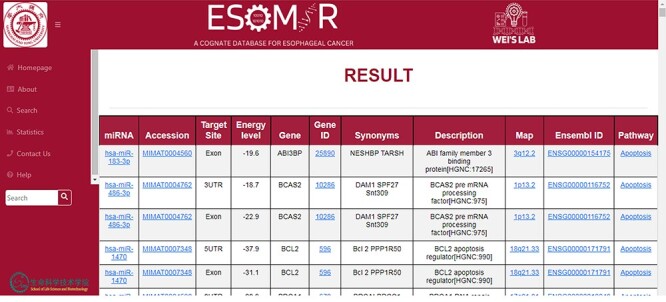
An illustration of the ESOMIR result page presented in a tabular format. The results show the miRNA, accession ID, target site, EL and other information including the gene name and pathways.

**Figure 7.**
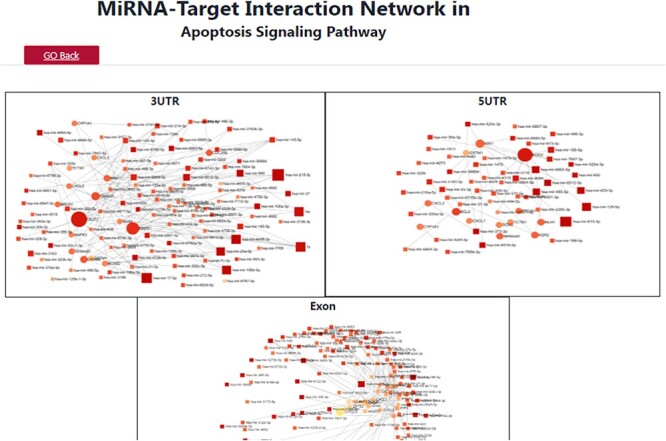
A depiction of the search results for the apoptosis signaling pathway, showcasing the target miRNA–mRNA network.

Furthermore, the table provides accessibility links to www.ensembl.org and www.biogps.org, allowing users to access the customizable layout through Ensembl ID and Entrez ID columns. The chromosome column provides the chromosomal location, with an accessible link to GeneCards, for users to obtain a broader vision of gene-centric information. The signaling pathways column maps the associated pathways of the specific miRNA, and users can also map the target gene onto the pathway in EC. The analysis includes vital pathways such as Wnt, NF-κB, TGFB, Apoptosis, Hippo, JAK-STAT and NOTCH. Users can access more detailed information about a specific pathway by being redirected to the KEGG pathway site. The resulting window provides pertinent information on crucial EC miRNAs and their well-recognized targets at various ELs. This information is deposited on the pathway information result page for users to access effortlessly. Target-based pathway information is available as an image file, which users can view in Figure 6. The target pathway search module presents pathway results that differ from other modules. It presents information about miRNA–mRNA with 3ʹUTR, 5′UTR and exon in a network with associated nodes and connections. The target pathway search module presents pathway results that differ from other modules. It presents information about miRNA–mRNA with 3′UTR, 5ʹUTR and exon in a network with associated nodes and connections as shown in Figure 7.

### The importance and abundance of data represented in ESOMIR

**Figure 8.**
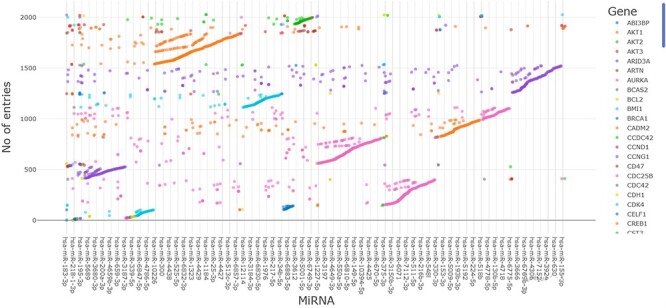
An illustration of >2000 mRNA–miRNA interactions that are exclusively linked to EC in the ESOMIR database.

The abundance and importance of data in the ESOMIR database are by its large number of entries, which reflects the large number of studies that have been investigated to collect, validate and verify miRNA–target interactions associated with EC. The database currently includes >2000 miRNA–target interactions curated from various sources, including experimental studies, computational predictions and literature mining. The density of data can be viewed in Figure 8.

**Figure 9.**
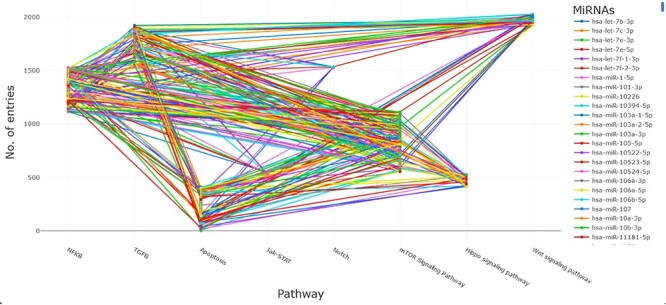
A depiction of the miRNA–target interactions, along with their corresponding signaling pathways.

The diverse information available in the ESOMIR database, including miRNA–target interactions along with their associated signaling pathways, is shown in Figure 9. This vast collection of information can be used to investigate the regulatory networks of miRNAs and their target genes in various biological processes, such as development, differentiation and disease. By utilizing the ESOMIR database, researchers can gain insight into the complex mechanisms underlying miRNA-mediated gene regulation and its role in disease pathogenesis, which could lead to the identification of potential therapeutic targets.

**Figure 10.**
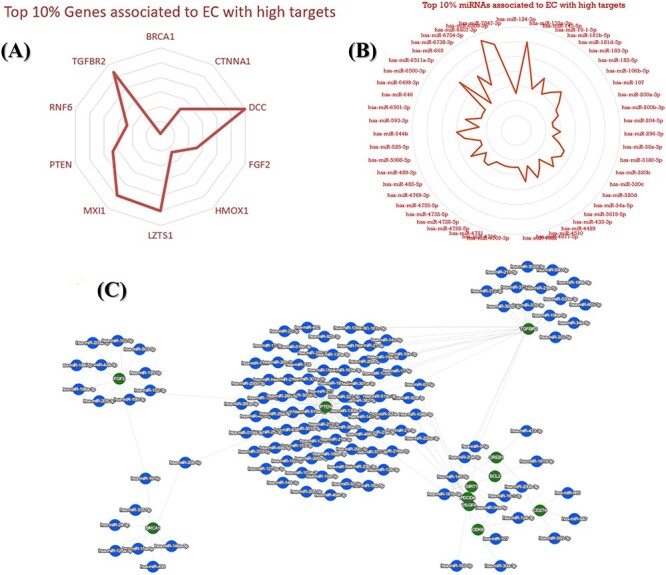
An illustration of top key mRNA and miRNA targets associated with EC. (**A**) The top 10% identified genes associated with EC. (**B**) The top 10% identified miRNAs associated with EC. (**C**) A network that visualizes the top connections and associations between mRNA and miRNAs, highlighting the most significant interactions among them.

Furthermore, we selected the key gene and key miRNA on the basis of binding site, rank score, number of targets and ELs. We illustrated the top 10% targeted/selected genes and miRNAs associated with EC. Few listed miRNAs are hsa-miR-6807-3p, hsa-miR-133a-3p, hsa-miR-6809-3p, hsa-miR-6867-5p, hsa-miR-6511a-5p, hsa-miR-92a-3p, hsa-miR-6738-3p, hsa-miR-7854-3p, hsa-miR-145-5p, hsa-miR-181b-5p, hsa-miR-183-5p, hsa-miR-544b, hsa-miR-6512-3p, hsa-miR-6758-5p, hsa-miR-6847-5p, hsa-miR-6893-5p, hsa-miR-106b-5p, hsa-miR-183-3p, hsa-miR-320b, hsa-miR-320c, hsa-miR-34a-5p, hsa-miR-107, hsa-miR-16-1-3p, hsa-miR-181d-5p, hsa-miR-200a-3p, hsa-miR-485-5p, hsa-miR-107, hsa-miR-124-3p, hsa-miR-200b-3p, hsa-miR-21-3p, hsa-miR-30a-3p, hsa-miR-92b-3p and hsa-miR-93-5p, and genes are BRCA1, CTNNA1, DCC, FGF2, HMOX1, LZTS1, MXI1, PTEN, RNF6 and TGFBR2. We illustrated the associations using a network as shown in Figure 10C. However, top 10% gene with highest score can be seen in Figure 10A and top 10% miRNA can be seen in Figure 10B.

## Discussion

Biological data have become a critical resource for studying complex diseases, with numerous databases available to target specific diseases and associated data. However, while miRNAs-based databases and their targets are essential, only some compile comprehensive knowledge about specific diseases and their targets. Currently, no available database exclusively provides information on the association of miRNAs with EC. To address this, we have developed ESOMIR, a dynamic and user-friendly database that provides comprehensive information on predicted and experimentally validated miRNAs with their putative targets. This database is anticipated to aid in the discovery of potential biomarkers for EC that possess diagnostic and therapeutic characteristics. Compared to other databases such as MicroCosm, miR2Disease, eMirit, TargetScan, HMDD v2.0 and miRCancer, ESOMIR contains the highest number of EC-specific miRNAs, with 877 humans (Figure 2A). This research also introduces novel miRNAs with their target information in different pathways. The ESOMIR database has a user-friendly web interface that enables the query of miRNAs, target mRNAs and their interaction network. Additionally, it provides target-ontology information for all genes in oncogenic pathways, making it a widespread platform for studying miRNA and its alleged role in the development and progression of EC. The identification and functional prediction of miRNA targets is essential in assessing the role of miRNAs in regulating or deregulating common genes and pathways that contribute to cancer development (67). Despite the continuous evolution of miRNA target prediction algorithms, effective prediction of miRNA targets in animals remains more challenging than in plants (68). miRNAs regulate target genes by binding to their 3ʹUTR, 5ʹUTR or coding regions through the seed region (5ʹ ends of miRNA), making it a more difficult task for target prediction tools because of incomplete base pairing at the complementary site (69). To minimize false positives, we employed a well-known target prediction algorithm, miRanda, RNA22v2 and DIANA-microR-CDS at different ELs, with validation by mirWalk and TargetScan. Targets predicted at the lowest EL 30 represent a strong pairing of miRNA to its target, whereas EL 15 is a more relaxed criterion for finding novel miRNA targets. This approach can help researchers narrow down and screen the most promising putative miRNA targets in acute lymphoblastic leukemia for validation. We found that the ESOMIR database contains 123 miRNA targets for humans, representing the highest number of predicted putative targets for EC miRNAs compared to any other available database (Figure 2B). Down-regulation of a wide range of oncogenes through miRNAs involved in different signaling pathways can maintain the healthy background of normal cells. Therefore, classifying EC miRNA targets based on different signaling pathways and interpreting miRNA–mRNA interaction networks at 3ʹUTR, 5ʹUTR and exon regions of target genes could provide detailed information on the role of miRNAs in EC initiation and progression. Several studies demonstrate the interaction between miRNA and signaling pathways during all procedures/hallmarks in EC. For instance, Li *et al.* reported cross-interaction between miRNA and NOTCH signaling in EC, specifically in ESCC (70). Moreover, Gao *et al.* demonstrated that the overexpression of miR-31 is an oncogene in ESCC, repressing the expression of LATS2 by the Hippo pathway and activating epithelial–mesenchymal transition (71).

According to the ESOMIR database, the interaction between miRNA–mRNA networks in different signaling pathways is crucial in facilitating cross talks, for example, miR-34a-5p inhibits proliferation, migration, invasion and epithelial–mesenchymal transition in ESCC by targeting LEF1 and inactivating the Hippo–YAP1/TAZ Signaling Pathway (72). miRNA-373 also promotes the development of ESCC by targeting LATS2 and OXR1. Furthermore, miRNA-21 promotes cell proliferation, migration and resistance to apoptosis in EC through the PTEN/PI3K/AKT signaling pathway (58, 73). The mTOR pathway and its interaction networks are identified as potential therapeutic targets in the treatment of EC (59). Researchers have identified the NF-κB signaling pathway as a transcriptional activator for a potential molecular marker for predicting and improving treatment efficacy in EC (74–76). The researchers have defined the roles of Wnt/β-catenin signaling pathway–related miRNAs in EC (64). They have found that down-regulation of miR-30a-3p/5p by activating the Wnt signaling pathway promotes cell proliferation in ESCC (77).

Various miRNAs are considered tumor suppressors or oncogenes in EC, including miR-10ab, miR-133ab, miR-145, miR-205, miR-99b, miR-199a-3p, miR-199a-5p, miR-126, miR-16-2, miR-15b, miR-143, miR-233, miR-486-5p and let-7a, as well as PDCD4, PTEN, ZEB1, Lzts, FGF2, DCC, MXI1, RNF6 and TGFB (78–87). The ESOMIR database provides information about these critical biomarkers and their associated targets. The functional annotation of miRNA targets at different cellular and molecular levels is essential for a systematic ontology of miRNAs. The ESOMIR database assigns ontology to targets of miRNAs and their association with biological processes at the cellular and molecular levels. Exploring relations between miRNA target genes and diseases could simplify our understanding of the pathogenesis of cancer and its progression. This information could help further understand the role of the miRNA cluster in regulating diverse pathways, leading to the development of novel therapeutic regimens for EC.

## Conclusion

The ESOMIR database is a valuable resource for researchers and clinicians interested in studying miRNA–target interactions associated with EC. With >2000 meticulously curated miRNA–target interactions, this database provides a wealth of information on the regulatory networks of miRNAs and their target genes in various biological processes. The database uses analysis and prediction methods, including experimentally endorsed miRNA(s) information. ESOMIR is a user-friendly interface that allows easy access to EC-associated data by searching for miRNAs, target genes, sequences, chromosomal positions and associated signaling pathways. The search modules are designed to provide specific data access to users based on their requirements. Additionally, the database provides information about network interactions, signaling pathways and region information of chromosomes associated with the 3ʹUTR or 5ʹUTR and exon sites. Overall, the ESOMIR database is a promising platform for identifying potential therapeutic targets and developing personalized cancer treatments. In addition to the current features and information in the ESOMIR database, future updates could include additional data on miRNA–target interactions associated with other types of cancers or diseases. These updates would further expand the database’s usefulness and make it a more comprehensive resource for researchers and clinicians. Additionally, incorporating machine learning algorithms could help to identify novel miRNA–target interactions and further enhance the predictive power of the database.

## Supplementary Material

baad063_Supp

## Data Availability

The data used to develop the database is attached as a supplementary file.

## References

[1] Sung H. , Ferlay J., Siegel R.L. et al. (2021) Global cancer statistics 2020: GLOBOCAN estimates of incidence and mortality worldwide for 36 cancers in 185 countries. *CA Cancer J. Clin.*, 71, 209–249. 33538338 10.3322/caac.21660

[2] Bandla S. , Pennathur A., Luketich J.D. et al. (2012) Comparative genomics of esophageal adenocarcinoma and squamous cell carcinoma. *Ann. Thorac. Surg.*, 93, 1101–1106. 22450065 10.1016/j.athoracsur.2012.01.064 PMC3401935

[3] Navab F. , Nathanson B.H. and Desilets D.J. (2015) The impact of lifestyle on Barrett’s esophagus: a precursor to esophageal adenocarcinoma. *Cancer Epidemiol.*, 39, 885–891. 26519660 10.1016/j.canep.2015.10.013

[4] Network C.G.A.R. et al. (2017) Integrated genomic characterization of oesophageal carcinoma. *Nature*, 541, 169–175. 28052061 10.1038/nature20805 PMC5651175

[5] Arnal M.J.D. , Arenas Á.F. and Arbeloa Á.L. (2015) Esophageal cancer: risk factors, screening and endoscopic treatment in western and eastern countries. *World J. Gastroenterol.*, 21, 7933–7943. 26185366 10.3748/wjg.v21.i26.7933 PMC4499337

[6] Griffiths-Jones S. (2007) Annotating noncoding RNA genes. *Annu. Rev. Genomics Hum. Genet.* 8, 279–298. 17506659 10.1146/annurev.genom.8.080706.092419

[7] Bartel D.P. (2009) MicroRNAs: target recognition and regulatory functions. *Cell*, 136, 215–233. 19167326 10.1016/j.cell.2009.01.002 PMC3794896

[8] Zhang B. , Pan X., Cobb G.P. et al. (2007) MicroRNAs as oncogenes and tumor suppressors. *Dev. Biol.*, 302, 1–12. 16989803 10.1016/j.ydbio.2006.08.028

[9] Lynam-Lennon N. , Maher S.G. and Reynolds J.V. (2009) The roles of microRNA in cancer and apoptosis. *Biol. Rev.*, 84, 55–71. 19046400 10.1111/j.1469-185X.2008.00061.x

[10] Tutar L. , Tutar E. and Tutar Y. (2014) MicroRNAs and cancer; an overview. *Curr. Pharm. Biotechnol.*, 15, 430–437. 24846068 10.2174/1389201015666140519095304

[11] Calin G.A. , Sevignani C., Dumitru C.D. et al. (2004) Human microRNA genes are frequently located at fragile sites and genomic regions involved in cancers. *Proc. Natl. Acad. Sci. U.S.A.*, 101, 2999–3004. 14973191 10.1073/pnas.0307323101 PMC365734

[12] Abbas G. and Krasna M. (2017) Overview of esophageal cancer. *Ann. Cardiothorac. Surg.*, 6, 131–136. 28447001 10.21037/acs.2017.03.03 PMC5387155

[13] Chen X. , Duan N., Zhang C. et al. (2016) Survivin and tumorigenesis: molecular mechanisms and therapeutic strategies. *J. Cancer*, 7, 314–323. 26918045 10.7150/jca.13332 PMC4747886

[14] Sethi S. , Ali S., Philip P.A. et al. (2013) Clinical advances in molecular biomarkers for cancer diagnosis and therapy. *Int. J. Mol. Sci.*, 14, 14771–14784. 23863689 10.3390/ijms140714771 PMC3742272

[15] Hassanein M. , Callison J.C., Callaway-Lane C. et al. (2012) The state of molecular biomarkers for the early detection of lung cancer. *Cancer Prevent. Res.*, 5, 992–1006. 10.1158/1940-6207.CAPR-11-0441 PMC3723112 22689914

[16] Tsuchiya N. , Sawada Y., Endo I. et al. (2015) Biomarkers for the early diagnosis of hepatocellular carcinoma. *World J. Gastroenterol.*, 21, 10573–10583. 26457017 10.3748/wjg.v21.i37.10573 PMC4588079

[17] Nangraj A.S. , Selvaraj G., Kaliamurthi S. et al. (2020) Integrated PPI- and WGCNA-retrieval of hub gene signatures shared between Barrett’s esophagus and esophageal adenocarcinoma. *Front. Pharmacol.*, 11, 1–14. 32903837 10.3389/fphar.2020.00881 PMC7438937

[18] Huang S. , Wei Y.K., Kaliamurthi S. et al. (2020) Circulating mir-1246 targeting UBE2C, TNNI3, TRAIP, UCHL1 genes and key pathways as a potential biomarker for lung adenocarcinoma: integrated biological network analysis. *J. Pers. Med.*, 10, 1–20. 10.3390/jpm10040162 PMC7712139 33050659

[19] Yang Z. , Yin H., Shi L. et al. (2020) A novel microRNA signature for pathological grading in lung adenocarcinoma based on TCGA and GEO data. *Int. J. Mol. Med.*, 45, 1397–1408. 32323746 10.3892/ijmm.2020.4526 PMC7138293

[20] Tang J. , Kong D., Cui Q. et al. (2018) Prognostic genes of breast cancer identified by gene co-expression network analysis. *Front. Oncol.*, 8, 374. 10.3389/fonc.2018.00374 PMC6141856 30254986

[21] He S. , Shi J., Mao J. et al. (2019) The expression of mir-375 in prostate cancer: a study based on GEO, TCGA data and bioinformatics analysis. *Pathol. Res. Pract.*, 215, 1–13. 30879885 10.1016/j.prp.2019.03.004

[22] Rajewsky N. and Socci N.D. (2004) Computational identification of microRNA targets. *Genome Biol.*, 5, 1–35. 10.1016/j.ydbio.2003.12.003 15013811

[23] Feber A. , Xi L., Luketich J.D. et al. (2008) MicroRNA expression profiles of esophageal cancer. *J. Thorac. Cardiovasc. Surg.*, 135, 255–260. 18242245 10.1016/j.jtcvs.2007.08.055 PMC2265073

[24] He B. , Yin B., Wang B. et al. (2012) MicroRNAs in esophageal cancer. *Mol. Med. Rep.*, 6, 459–465. 22751839 10.3892/mmr.2012.975

[25] Harada K. , Baba Y., Ishimoto T. et al. (2016) The role of microRNA in esophageal squamous cell carcinoma. *J. Gastroenterol.*, 51, 520–530. 26794004 10.1007/s00535-016-1161-9

[26] Gao R. , WangZ., LiuQ. et al. (2020) MicroRNA-105 plays an independent prognostic role in esophageal cancer and acts as an oncogene. *Cancer Biomark.*, 27, 173–180.3179666310.3233/CBM-180

[27] Garman K.S. , Owzar K., Hauser E.R. et al. (2013) MicroRNA expression differentiates squamous epithelium from Barrett’s esophagus and esophageal cancer. *Dig. Dis. Sci.*, 58, 3178–3188. 23925817 10.1007/s10620-013-2806-7 PMC4180409

[28] Griffiths-Jones R.J. , Grocock S., Van Dongen A. et al. (2006) Enright, miRBase: microRNA sequences, targets and gene nomenclature. *Nucleic Acids Res.*, 34, D140–D144. 16381832 10.1093/nar/gkj112 PMC1347474

[29] Georgakilas G. , Vlachos I.S., Zagganas K. et al. (2016) DIANA-miRGen v3.0: accurate characterization of microRNA promoters and their regulators. *Nucleic Acids Res.*, 44, D190–D195. 26586797 10.1093/nar/gkv1254 PMC4702888

[30] Cho S. , Jang I., Jun Y. et al. (2012) Mirgator v3.0: a microRNA portal for deep sequencing, expression profiling and mRNA targeting. *Nucleic Acids Res.*, 41, D252–D257. 23193297 10.1093/nar/gks1168 PMC3531224

[31] Sticht C. , De La Torre C., Parveen A. et al. (2018) miRWalk: an online resource for prediction of microRNA binding sites. *PLoS One*, 13, 1–6. 10.1371/journal.pone.0206239 PMC6193719 30335862

[32] Huang H.Y. , Lin Y.C.D., Li J. et al. (2020) miRTarBase 2020: updates to the experimentally validated microRNA–target interaction database. *Nucleic Acids Res.*, 48, D148–D154. 31647101 10.1093/nar/gkz896 PMC7145596

[33] Hsu P.W. , Huang H.D., Hsu S.D. et al. (2006) miRNAMap: genomic maps of microRNA genes and their target genes in mammalian genomes. *Nucleic Acids Res.*, 1, D135–D139. 10.1093/nar/gkj135 PMC1347497 16381831

[34] Xiao F. , Zuo Z., Cai G. et al. (2009) miRecords: an integrated resource for microRNA–target interactions. *Nucleic Acids Res.*, 37, D105–D110. 18996891 10.1093/nar/gkn851 PMC2686554

[35] Agarwal V. , Bell G.W., Nam J.W. et al. (2015) Predicting effective microRNA target sites in mammalian mRNAs. *eLife*, 4, e05005. 10.7554/eLife.05005 PMC4532895 26267216

[36] Wong N. and Wang X. (2015) miRDB: an online resource for microRNA target prediction and functional annotations. *Nucleic Acids Res.*, 43, D146–D152. 25378301 10.1093/nar/gku1104 PMC4383922

[37] Krek A. , Grün D., Poy M.N. et al. (2005) Combinatorial microRNA target predictions. *Nat. Genet.*, 37, 495–500. 15806104 10.1038/ng1536

[38] Quillet A. , Saad C., Ferry G. et al. (2020) Improving bioinformatics prediction of microRNA targets by ranks aggregation. *Front. Genet.*, 10, 1–14. 10.3389/fgene.2019.01330 PMC6997536 32047509

[39] Khurana R. , Verma V.K., Rawoof A. et al. (2014) OncomiRdbB: a comprehensive database of microRNAs and their targets in breast cancer. *BMC Bioinform.*, 15, 1–12. 10.1186/1471-2105-15-15 PMC3926854 24428888

[40] Xie B. , Ding Q., Han H. et al. (2013) miRCancer: a microRNA–cancer association database constructed by text mining on literature. *Bioinformatics*, 29, 638–644. 23325619 10.1093/bioinformatics/btt014

[41] Tan H. , Kim P., Sun P. et al. (2021) miRactDB characterizes miRNA–gene relation switch between normal and cancer tissues across pan-cancer. *Brief. Bioinform.*, 22, bbaa089. 10.1093/bib/bbaa089 PMC8138827 32436932

[42] Ruepp A. , Kowarsch A., Schmidl D. et al. (2010) PhenomiR: a knowledgebase for microRNA expression in diseases and biological processes. *Genome Biol.*, 11, 1–11. 10.1186/gb-2010-11-1-r6 PMC2847718 20089154

[43] Li Y. , Qiu C., Tu J. et al. (2014) HMDD v2.0: a database for experimentally supported human microRNA and disease associations. *Nucleic Acids Res.*, 42, D1070–D1074. 24194601 10.1093/nar/gkt1023 PMC3964961

[44] Sarver A.L. , Phalak R., Thayanithy V. et al. (2010) S-MED: sarcoma microRNA expression database. *Lab. Invest.*, 90, 753–761. 20212452 10.1038/labinvest.2010.53

[45] Jiang Q. , Wang Y., Hao Y. et al. (2009) mir2disease: a manually curated database for microRNA deregulation in human disease. *Nucleic Acids Res.*, 37, D98–D104. 18927107 10.1093/nar/gkn714 PMC2686559

[46] Lu T.P. , Lee C.Y., Tsai M.H. et al. (2012) miRSystem: an integrated system for characterizing enriched functions and pathways of microRNA targets. *PLoS One*, 7, 1–14. 10.1371/journal.pone.0042390 PMC3411648 22870325

[47] Forbes S. , Clements J., Dawson E. et al. (2006) COSMIC 2005. *Br. J. Cancer*, 94, 318–322. 16421597 10.1038/sj.bjc.6602928 PMC2361125

[48] Kanehisa M. and Goto S. (2000) KEGG: Kyoto Encyclopedia of Genes and Genomes. *Nucleic Acids Res.*, 28, 27–30. 10592173 10.1093/nar/28.1.27 PMC102409

[49] Essack M. , Radovanovic A., Schaefer U. et al. (2009) DDEC: Dragon database of genes implicated in esophageal cancer. *BMC Cancer*, 9, 1–7. 19580656 10.1186/1471-2407-9-219 PMC2711974

[50] Kato K. , Yamashita R., Matoba R. et al. (2005) Cancer gene expression database (CGED): a database for gene expression profiling with accompanying clinical information of human cancer tissues. *Nucleic Acids Res.*, 33, D533–D536. 15608255 10.1093/nar/gki117 PMC540071

[51] Piñero J. , Ramírez-Anguita J.M., Saüch-Pitarch J. et al. (2020) The DisGeNET knowledge platform for disease genomics: 2019 update. *Nucleic Acids Res.*, 48, D845–D855. 31680165 10.1093/nar/gkz1021 PMC7145631

[52] Kinsella R.J. , Kähäri A., Haider S. et al. (2011) Ensembl BioMarts: a hub for data retrieval across taxonomic space. *Database*, 2011, bar030. 10.1093/database/bar030 PMC3170168 21785142

[53] Miranda K.C. , Huynh T., Tay Y. et al. (2006) A pattern-based method for the identification of microRNA binding sites and their corresponding heteroduplexes. *Cell*, 126, 1203–1217. 16990141 10.1016/j.cell.2006.07.031

[54] Paraskevopoulou M.D. , Georgakilas G., Kostoulas N. et al. (2013) DIANA-microT web server v5.0: service integration into miRNA functional analysis workflows. *Nucleic Acids Res.*, 41, W169–W173. 23680784 10.1093/nar/gkt393 PMC3692048

[55] You Z. , Xu D., Ji J. et al. (2012) JAK/STAT signal pathway activation promotes progression and survival of human oesophageal squamous cell carcinoma. *Clin. Transl. Oncol.*, 14, 143–149. 22301404 10.1007/s12094-012-0774-6

[56] Meng X. , Lu P., Mei J. et al. (2014) Expression analysis of miRNA and target mRNAs in esophageal cancer. *Braz. J. Med. Biol. Res.*, 47, 811–817. 25098614 10.1590/1414-431X20143906 PMC4143210

[57] Cheng Z. , Geng H., Cheng Y. et al. (2018) Effects of MiR-210 on proliferation, apoptosis and invasion abilities of esophageal cancer cells. *J. BUON*, 23, 814–819. 30003756

[58] Javadinia S.A. , Shahidsales S., Fanipakdel A. et al. (2018) The esophageal cancer and the PI3K/AKT/mTOR signaling regulatory microRNAs: a novel marker for prognosis, and a possible target for immunotherapy. *Curr. Pharm. Des.*, 24, 4646–4651. 30636576 10.2174/1381612825666190110143258

[59] Akbarzadeh M. , Mihanfar A., Akbarzadeh S. et al. (2021) Crosstalk between miRNA and PI3K/AKT/mTOR signaling pathway in cancer. *Life Sci.*, 285, 1–14. 10.1016/j.lfs.2021.119984 34592229

[60] Yang L. , Sun K., Chu J. et al. (2018) Long non-coding RNA FTH1P3 regulated metastasis and invasion of esophageal squamous cell carcinoma through SP1/NF-kB pathway. *Biomed. Pharmacother.*, 106, 1570–1577. 30119232 10.1016/j.biopha.2018.07.129

[61] Wang L. , Wang L., Chang W. et al. (2019) MicroRNA-373 promotes the development of esophageal squamous cell carcinoma by targeting *LATS2* and *OXR1*. *Int. J. Biol. Markers*, 34, 148–155. 30852977 10.1177/1724600819827964

[62] Zhang Y. , Pan T., Zhong X. et al. (2014) Nicotine upregulates microRNA-21 and promotes TGF-*β*-dependent epithelial-mesenchymal transition of esophageal cancer cells. *Tumor Biol.*, 35, 7063–7072. 10.1007/s13277-014-1968-z 24756761

[63] Jia Y. , Yang Y., Zhan Q. et al. (2012) Inhibition of SOX17 by microRNA 141 and methylation activates the WNT signaling pathway in esophageal cancer. *J. Mol. Diagn.*, 14, 577–585. 22921431 10.1016/j.jmoldx.2012.06.004

[64] Chu Y. , Wang R. and Liu X.-L. (2022) Roles of Wnt/*β*-catenin signaling pathway related microRNAs in esophageal cancer. *World J. Clin. Cases*, 10, 2678–2686. 35434118 10.12998/wjcc.v10.i9.2678 PMC8968815

[65] Subramaniam D. , Ponnurangam S., Ramamoorthy P. et al. (2012) Curcumin induces cell death in esophageal cancer cells through modulating notch signaling. *PLoS One*, 7, 1–11. 10.1371/journal.pone.0030590 PMC3281833 22363450

[66] Roychowdhury D. , Gupta S., Qin X. et al. (2021) emiRIT: a text-mining-based resource for microRNA information. *Database*, 2021, 1–20. 10.1093/database/baab031 PMC8163238 34048547

[67] Ritchie W. , Flamant S. and Rasko J.E. (2009) Predicting microRNA targets and functions: traps for the unwary. *Nat. Methods*, 6, 397–398. 19478799 10.1038/nmeth0609-397

[68] Lewis B.P. , Shih I.H., Jones-Rhoades M.W. et al. (2003) Prediction of mammalian microRNA targets. *Cell*, 115, 787–798. 14697198 10.1016/s0092-8674(03)01018-3

[69] Xu W. , San Lucas A., Wang Z. et al. (2014) Identifying microRNA targets in different gene regions. *BMC Bioinform.*, 15, 1–11. 10.1186/1471-2105-15-S7-S4 PMC4110731 25077573

[70] Li Y. , Li Y. and Chen X. (2021) NOTCH and esophageal squamous cell carcinoma. *Adv Exp Med Biol*, 1287, 59–68. 33034026 10.1007/978-3-030-55031-8_5 PMC7895477

[71] Gao Y. , Yi J., Zhang K. et al. (2017) Downregulation of MiR-31 stimulates expression of LATS2 via the hippo pathway and promotes epithelial-mesenchymal transition in esophageal squamous cell carcinoma. *J. Exp. Clin. Cancer Res.*, 36, 1–20. 29145896 10.1186/s13046-017-0622-1 PMC5689139

[72] Wang X. , Zhao Y., Lu Q. et al. (2020) MiR-34a-5p inhibits proliferation, migration, invasion and epithelial-mesenchymal transition in esophageal squamous cell carcinoma by targeting lef1 and inactivation of the Hippo-YAP1/TAZ signaling pathway. *J. Cancer*, 11, 3072–3081. 32226522 10.7150/jca.39861 PMC7086260

[73] Wu Y.R. , Qi H.J., Deng D.F. et al. (2016) MicroRNA-21 promotes cell proliferation, migration, and resistance to apoptosis through PTEN/PI3K/AKT signaling pathway in esophageal cancer. *Tumor Biol.*, 37, 12061–12070. 10.1007/s13277-016-5074-2 27188433

[74] Izzo J. , Wu T., Malhotra U. et al. (2006) Transcription factor NFkB a potential molecular marker for predicting and improving treatment efficacy in esophageal cancer. *J. Clin. Oncol.*, 24, 10065–10065.

[75] Li J. , Wang K., Chen X. et al. (2012) Transcriptional activation of microRNA-34a by NF-kappa B in human esophageal cancer cells. *BMC Mol. Biol.*, 13, 1–10. 22292433 10.1186/1471-2199-13-4 PMC3311059

[76] Naganuma S. , Whelan K.A., Natsuizaka M. et al. (2012) Notch receptor inhibition reveals the importance of cyclin D1 and Wnt signaling in invasive esophageal squamous cell carcinoma. *Am. J. Cancer Res.*, 2, 459–475. 22860235 PMC3410579

[77] Qi B. , Wang Y., Chen Z.J. et al. (2017) Down-regulation of miR-30a-3p/5p promotes esophageal squamous cell carcinoma cell proliferation by activating the Wnt signaling pathway. *World J. Gastroenterol.*, 23, 7965–7977. 29259372 10.3748/wjg.v23.i45.7965 PMC5725291

[78] Kano M. , Seki N., Kikkawa N. et al. (2010) miR-145, miR-133a and miR-133b: tumor-suppressive miRNAs target FSCN1 in esophageal squamous cell carcinoma. *Int. J. Cancer*, 127, 2804–2814. 21351259 10.1002/ijc.25284

[79] Matsushima K. , Isomoto H., Kohno S. et al. (2010) MicroRNAs and esophageal squamous cell carcinoma. *Digestion*, 82, 138–144. 20588024 10.1159/000310918

[80] Ma W.J. , Lv G.D., Tuersun A. et al. (2011) Role of microRNA-21 and effect on PTEN in Kazakh’s esophageal squamous cell carcinoma. *Mol. Biol. Rep.*, 38, 3253–3260. 21104017 10.1007/s11033-010-0480-9

[81] Matsushima K. , Isomoto H., Yamaguchi N. et al. (2011) miRNA-205 modulates cellular invasion and migration via regulating zinc finger E-box binding homeobox 2 expression in esophageal squamous cell carcinoma cells. *J. Transl. Med.*, 9, 1–12. 10.1186/1479-5876-9-30 PMC3076245 21426561

[82] Vecchione A. , Croce C.M. and Baldassarre G. (2007) Fez1/Lzts1 a new mitotic regulator implicated in cancer development. *Cell Div.*, 2, 1–5. 17718912 10.1186/1747-1028-2-24 PMC2075490

[83] Shi H. , Xu J., Zhao R. et al. (2016) FGF2 regulates proliferation, migration, and invasion of ECA109 cells through PI3K/Akt signalling pathway in vitro. *Cell Biol. Int.*, 40, 524–533. 26833879 10.1002/cbin.10588

[84] Lui Park H. , Sook Kim M., Yamashita K. et al. (2008) DCC promoter hypermethylation in esophageal squamous cell carcinoma. *Int. J. Cancer*, 122, 2498–2502. 18302152 10.1002/ijc.23434

[85] Li X.J. , Wang D.Y., Zhu Y. et al. (1999) Mxi1 mutations in human neurofibrosarcomas. *Jpn. J. Cancer Res.*, 90, 740–746. 10470286 10.1111/j.1349-7006.1999.tb00809.x PMC5926139

[86] Hu N. , Wang C., Hu Y. et al. (2005) Genome-wide association study in esophageal cancer using GeneChip mapping 10k array. *Cancer Res.*, 65, 2542–2546. 15805246 10.1158/0008-5472.CAN-04-3247

[87] Jin G. , Deng Y., Miao R. et al. (2008) TGFB1 and TGFBR2 functional polymorphisms and risk of esophageal squamous cell carcinoma: a case–control analysis in a Chinese population. *J. Cancer Res. Clin. Oncol.*, 134, 345–351. 17680270 10.1007/s00432-007-0290-1 PMC12161658

